# Treatment for acute flares of gout: A protocol for systematic review

**DOI:** 10.1097/MD.0000000000019668

**Published:** 2020-04-03

**Authors:** Hongzhi Tang, Guixing Xu, Qianhua Zheng, Ying Cheng, Hui Zheng, Juan Li, Zihan Yin, Fanrong Liang, Jiao Chen

**Affiliations:** a Outpatient department of Sichuan orthopedic hospital; b The Acupuncture and Tuina School, The 3rd Teaching Hospital, Chengdu University of Traditional Chinese Medicine; c The First Affiliated Hospital of Chengdu University of Chinese Medicine, Chengdu, Sichuan, China.

**Keywords:** gout, network meta-analysis, protocol, systematic review

## Abstract

**Introduction:**

The current evidence confirms the effectiveness and safety of several drug interventions in the treatment of acute flares of gout, however, the most preferred drugs are still unclear. We, therefore, seek to conduct a network meta-analysis that can systematically compare non-steroidal anti-inflammatory drugs (NSAIDs), COXIBs, colchicine, hormones, or IL-1 receptor antagonists, etc. for acute gout based on the latest evidence.

**Methods and analysis:**

Nine online databases are searched with inception to September 1, 2019; there will be no language restrictions on the included trials. Randomized controlled trials that include patients with acute flares of gout receiving drug therapy versus a control group will be included. The selection of studies, risk of bias assessment and data extraction will be conducted by 2 independent researchers. Bayesian network meta-analysis is applied using the Markov chain Monte Carlo method with Stata or R. The dichotomous data will be presented as risk ratios with 95% CIs and the continuous data will be presented as weighted mean differences or standardized mean differences with 95% CIs. Evidence quality will be evaluated using the GRADE system.

**Ethics and dissemination:**

This network meta-analysis will not involve private information from personal or imperil their rights, so, ethical approval is not required. The results of this network meta-analysis may be published in a journal or publicized in concerned conferences.

## Abstract 2

Supplemental Digital Content is available in the text

**Strengths and Limitations of this study**

- To the best of our knowledge, this is the first network meta-analysis protocol to assess the effectiveness and safety of drug therapies for adult patients with acute flares of gout.
- The results of this network review will be beneficial to clinicians in making decisions the optimal method of treating the disease, and help patients with acute flares of gout seeking optimal treatment.
- Patient comorbidities often limit the choice of treatment for acute gout.
- Controversial because of its indirectness and complexity.

## 1 Introduction

Gouty arthritis is a group of diseases caused by decreased uric acid excretion. Its clinical syndromes include hyperuricemia, tophi deposits, recurrent acute arthritis, and tophus chronic arthritis. And the joints often become subtle and red, swollen, with severe pain, malfunction joint activities, and decreased quality of life of patients, and it can affect the kidneys in severe cases. Studies have shown that the incidence of gout has increased in the past few decades,^[1]^ with prevalence rates from 1.7% to 3.8% in all country.^[2–5]^ The purpose of gout treatment is to quickly and effectively relieve and eliminate acute symptoms, to prevent the recurrence of acute arthritis, reduce blood uric acid, and eliminate the cause.^[6]^ At present, the modern medical clinical guidelines recommend non-steroidal anti-inflammatory drugs (NSAIDs), COXIBs, colchicine, hormones, or IL-1 receptor antagonists, etc. treatment in the acute phase, and active uric acid decreasing treatment in the chronic phase and intermittent phase to prevent the recurrence of gouty arthritis.^[7–9]^ The systematic reviews/meta-analysis published so far only compared between any 2 drug therapies, but without recommendation or evidence for the most optimal therapy.^[5,7,9–15]^ Meanwhile, the guidelines of the American College of Rheumatology in 2016 only suggest that physicians should combine the severity of gout attacks, the number and duration of affected joints, the patient's preferences, previous treatment responses, and comorbidities in the acute phase of gout.^[9]^ Hence it did not recommend the priority level of drugs.^[9]^ And the guidelines of the European Union Against Rheumatism in 2016 also recommended that the treatment of the acute phase of gout should depend on the severity, number of joints involved and duration.^[7,8]^ Although the treatment of early use of low-dose colchicine is proposed, it is still unclear whether the early use of low-dose colchicine is superior to NSAID. Further, some studies found that more than 90% of patients had at least one contraindication to NSAIDs and that about one-third of patients prescribed colchicine had at least one major contraindication.^[16,17]^ Therefore, the evidence of the optimal drug therapy for acute episodes of gout is unidentified.

We hence seek to conduct a network meta-analysis that can systematically compare multiple drug intervention therapies for acute gout based on the latest evidence.

## 2 Methods

### 2.1 Criteria for inclusion

#### 2.1.1 Types of studies

Randomized controlled trials (RCT), restricted to English and Chinese, will be considered applicable. RCTs using single-blind, double-blind, or open-label design are included. By defining double-blind, we meant that both participants and RCTs blinding outcome assessors or statisticians instead of participants and care providers are classified as open-label trials. Multiple arms trials met the above criteria are included. RCTs with crossover design or n-of-1 design are excluded. For crossover trials, data are extracted from the first period only, to avoid potential carryover effects.

#### 2.1.2 Types of participants

Adult patients (aged ≥18 years) diagnosed with acute flares of gout according to the American College of Rheumatology or the European League Against Rheumatism preliminary criteria are included, with the onset of pain <48 hours before enrollment in the research.

#### 2.1.3 Types of interventions

Pharmacologic treatments, such as NSAIDs, COXIBs, colchicine, hormones, or IL-1 receptor antagonists, etc. for managing acute gout include drugs used in clinical practice at the time of the study and new drugs under investigation. Generic pain relief medication or alternative and complementary therapies such as acupuncture or collagen are excluded.

#### 2.1.4 Types of comparator(s)/control

Trials with positive comparators and placebo control groups are included.

#### 2.1.5 Types of outcome measures

We assess the outcome indicators based on a systematic review^[18]^ in this study.

#### 2.1.6 Primary outcomes

1. Pain: Pain scores are measured with visual analogue scale (VAS), numerical rating scale or Likert scale.^[18]^

#### 2.1.7 Secondary outcomes

1. Inflammation reaction (joint swelling, erythema, tenderness): if an individual trial is reported with more than 1 parameter, we extracted only 1 according to the following hierarchy: swelling, erythema, tenderness. Where applicable, we extracted data both in an index joint and as the total number of inflamed joints.^[18]^
2. Patient global assessment of response to therapy (PGART).^[18]^
3. Health-related quality of life (HRQoL): as reported by generic questionnaires (such as the 36-item Short Form (SF-36)) or by disease-specific questionnaires (such as the Gout Assessment Questionnaire (GAQ) or Gout Impact Scale (GIS)).
4. The function of joints: the improvement of function is assessed by the Health Assessment Questionnaire (HAQ) or by any other method. We consider that disability and activity limitations are comparable concepts to function, so, we regard the function of joints as secondary outcomes.^[18]^
5. The number of withdrawals due to adverse events (AE) and serious adverse events (SAE).

We extract outcomes at all time points measured in the included trials. We plan to pool available data into short-term (up to 2 weeks), medium-term (2–6 weeks) and long-term (more than 6 weeks) outcomes, when data are available.

### 2.2 Search methods for identification of studies

#### 2.2.1 Electronic searches

From the inception dates to September 1, 2019, the following databases will be searched: EMBASE, Ovid, the Cochrane Library, MEDLINE, PubMed, Web of Science, Chinese Biomedical Literature Database (CBM), Chinese National Knowledge Infrastructure (CNKI), Wanfang Database, the Chongqing VIP (VIP). The searching strategy of PubMed is presented in Supplemental Digital Content (Appendix 1).

#### 2.2.2 Searching other resources

Ongoing trials with unpublished data will also be retrieved from the following clinical trial registries: the NIH clinical registry Clinical Trials. The International Clinical Trials Registry Platform (ICTRP), the Australian New Zealand Clinical Trials Registry and the Chinese clinical registry. Additional trials will be further identified according to the list of all identified publications including relevant systematic reviews and meta-analyses. Useful but incomplete data will be obtained for data synthesis from the contact trial researcher.

### 2.3 Data collection and analysis

#### 2.3.1 Selection of studies

Before the selection of publications, a program for screening will be counseled and developed among all the reviewers. After electronic searches, the results will be exported to a database is called “gout” created by Endnote software (version X9). Publications obtained from other sources will also be imported to the same database. Two reviewers (THZ and YZH) will independently screen out the titles and abstracts in this database according to the criterion below: first, find out and delete the duplicates (same content in different languages or different published forms such as journal articles and conference abstracts, or 2 articles wrote the same trial from different aspects); second, exclude studies in which only non-pharmacological treatment are received in an experimental group or participants are diagnosed with other severe conditions(such as severe nephropathy, heart diseases, cancer and so on); third, None RCT with parallel design will be excluded; fourth, studies including participants under the age of 18 will be excluded. If the reviewers (THZ and YZH) are not able to screen the studies based on their titles and abstracts, 2 other reviewers (XGX and ZQH) will screen the full text of these studies. When disagreements occur between reviewers, they will be resolved through discussion. If no consensus reaches, a third reviewer (CY) will be consulted. The process of studies screen is presented in Supplemental Digital Content (Appendix 2).

#### 2.3.2 Data extraction and management

Before data extraction, a standardized data extraction form will be developed by the meeting of all reviewers. Then we will use this form to extract information from at least 3 studies to check its feasibility. Two reviewers (XGX and YZH) will extract the following information from the database: organizational information (including year of publication, reference ID, reviewers name, the first author of the study, publication source, etc.), design of trial (design of the study, number of groups and participants for treatment and control, method of randomization, method of analysis, blinding, objectives of the study, etc.), participants (age, gender, ethnicity, country, diagnosis, duration, etc.), interventions and controls (method of the intervention, number of treatment, frequency of treatment, duration of a session, name and type for control, information of caring, additional treatment, etc.), outcome measurements (primary outcome and secondary outcome according to types of outcome measures, timeline for assessment, length of follow-up, etc.), results (mean, SD, observed events after intervention, total sample size, etc.) etc. The disagreement between the 2 reviewers will be solved by discussion among all the reviewers. The extraction data will be listed in Excel2016, and other reviewers (LJ) will check the data entered to ensure the consistency and correct data entry errors.

#### 2.3.3 Assessment of risk of bias in included studies

The quality of the included trials will be evaluated by 2 reviewers (CY and ZH) using the Cochrane Collaborations tool.^[19]^ Six aspects (randomly generated sequence number, allocation concealment, blinding of participants and personnel, blinding of outcome assessment, incomplete outcome data, selective reporting, and other bias when required) will be assessed. For each aspect, the trial will be rated as high, low risk, or unclear of bias. A trial that is rated high risk of bias in 1 or more aspects will be graded as “high risk”, while a low risk of bias in all aspects will be graded as “low risk”. If there is a low or unclear risk of bias for all main aspects, the trial will be rated as “unclear risk”. The contact person or corresponding author will be contacted if basic information is missing for the risk of bias assessment. The rating results will be cross-checked and discrepancies resolved through discussions and the arbitration of a third reviewer (CJ).

#### 2.3.4 Measures of treatment effect

Efficacy data will be synthesized and statistically analyzed in R3.5.1. Dichotomous data will be investigated by using a risk ratio with 95% CIs. For continuous outcomes, data will be analyzed by using a standard mean difference (SMD) with 95% CIs or a weighted mean difference (WMD). The WMD will be used for the same scale or the same assessment instrument; SMD will be used for different assessment tools.

#### 2.3.5 Unit of analysis issues

The units of each outcome from different trials will be converted to the International System of Units before statistical analysis.

#### 2.3.6 Dealing with missing data

The authors of included studies with missing data will be contacted to get data. If the missing data is not accessible, we will exclude these articles and synthesis the rest of the included studies.

#### 2.3.7 Assessment of consistency

A consistency examination will be taken using the Z test. We will calculate the *P* value to find out whether there are inconsistencies among the comparison of direct and indirect. If the *P* > .05, there is no statistical significance, so the comparison of direct and indirect is consistency; on the contrary, inconsistency is considered.^[33]^

#### 2.3.8 Data synthesis

Pragmatic trials, with patients treatments, shift between modalities and dosages according to treatment response, are investigated in a narrative synthesis. Other data synthesis will be conducted (http://www.r-project.org). We defined Sham interventions and placebo as inert control. Network meta-analysis including both direct and indirect evidence was performed by using a Bayes method. SMDs and RRs of network meta-analysis were also computed along with their 95% CIs.

The reliability of the result of network meta-analysis mainly depends on the transitivity of the evidence. The transitivity was usually defined as the similarity level in effect modifiers (eg, study design, the severity of illness at baseline, treatment dose, and study quality).^[20]^

We will assess the transitivity of the network largely in the consistency between direct and indirect analysis.^[21]^ Consistency of the network meta-analysis will be estimated by the Z test to explore the difference between direct and indirect estimates. The contribution of different designs to the final effect size of the network meta-analysis will be evaluated by net-heat plots.

The drug therapies will be ranked by using P-score that measures the extent of certainty when treatment is better than control. A P-score equals 100% when a treatment is certain to be the best and 0% of a P-score indicates a treatment to be the worst.^[22]^

#### 2.3.9 Sensitive analysis or Subgroup analysis or meta-regression

If enough trials are included, we will explore the following potential sources of inconsistency using sensitive analysis or subgroup analyses or meta-regression:

1. Studies with low risk of bias compared to trials with a high risk of bias;
2. Whether there is health education^[7]^;
3. Gout severity according the Gout Impact Scale (GIS).^[7,32]^
4. We will conduct a subgroup analysis according the patient comorbidities to make the results more suitable for clinical.
5. We will assess a subgroup analysis from variant nationalities to check the applicability for local people.^[23]^

#### 2.3.10 Evidence quality evaluation

Two reviewers will use the Grading of Recommendations Assessment, Development and Evaluation (GRADE) system to independently assess the quality of evidence for each outcome24. Evidence quality will be rated “high”, “moderate”, “low” or “very low” according to the GRADE rating standards. The quality of evidence of a specific study will be assessed according to the risk of bias, inconsistency, indirectness, imprecision, publication bias, large effect, dose-response, and all plausible confounding.^[24,25]^ A summary of findings table will be generated and included in the final report.^[25]^

#### 2.3.11 Ethics and dissemination

This review does not require ethical approval duo to data that we will not endanger the individuals privacy or compromise their rights. The results of a review that provide systematically view and evidence of drug therapies for acute gout will also give implication for clinical practice and further research, and the founding of this study may be published in a peer-reviewed journal or distributed at relevant conferences.

## 3 Discussion

In this report, we elucidate a protocol for network meta-analysis of using drug therapy to treat acute gout, which is a prevalent public health problem. We use network meta-analysis for direct and indirect evaluation and comparison of evidence, even if the 2 treatments have never been directly compared before. The analysis can summarize a series of randomized clinical trial data for different treatment outcomes, and then point to a given treatment endpoint for Confidence interval estimation while assessing its relevance.^[26]^

Although we have conducted similar studies on before,^[27,28]^ we still encounter problems. The major concern is how to identify all clinical drug. After consultation with experts, the decision is made and the inclusion of the study is determined primarily by 2 aspects. First, the initially planned drug is determined according to the gout suppressants of MeSH term of PubMed and systematic review/meta-analysis and guidelines.^[7,29,30]^ And according to the appropriate addition and subtraction by the clinical expert, the second difficulty in this study was to identify the inclusion population. We followed the American College of Rheumatology or the European League Against Rheumatism preliminary criteria, comply with the Chinese Gout Guidelines,^[29,31]^ and limited the number of patients enrolled to 48 hours after the onset of gout. The method of dealing with missing data, in this protocol, is also a major issue. Four options are provided in the Cochrane handbook, about how to deal with missing data. After discussion, we will try to contact the authors to get the missing data first, if the missing data is not accessible, we will exclude it to avoid bias to the results.

This network meta-analysis will give a summary of the current evidence on the effectiveness and safety of drug therapies for patients with acute gout and the assessment of evidence quality based on GRADE. This network meta-analysis result will benefit patients with acute gout and clinician for evidence of optimal treatment options.

## Author contributions

THZ, XGX, and LFR contributed to the conception and design of the study protocol. The search strategy was developed and run by YZH and THZ, who will also screen the title and abstract of the studies after running the search strategy. XGX and ZQH will screen full copies of the remaining studies after the title and abstract selection. YZH and XQW will extract information from the included studies and enter into the electronic database. LJ will check the accuracy and completeness of the data entry. CJ and ZH will give analysis suggestions during data synthesis. All the authors drafted and revised this study protocol and approved it for publication.

## Supplementary Material

Supplemental Digital Content

## Supplementary Material

Supplemental Digital Content
