# Supplementary material for: Treatment for acute flares of gout: A protocol for systematic review
Source: Medicine (Baltimore). 2020 Apr 3;99(14):e19668. doi: 10.1097/MD.0000000000019668 (PMC7440275; doi:10.1097/MD.0000000000019668)
Supplement: Supplemental Digital Content [file medi-99-e19668-s001.docx]

| **AppendixⅠ Search strategy used in Pubmed database** | |
| --- | --- |
| **Number** | **Search terms** |
| 1 | randomized controlled trial [All Fields] |
| 2 | controlled clinical trial [All Fields] |
| 3 | randomized [All Fields] |
| 4 | randomised [All Fields] |
| 5 | placebo [All Fields] |
| 6 | randomly [All Fields] |
| 7 | trial [All Fields] |
| 8 | groups [All Fields] |
| 9 | or/1-8 |
| 10 | hyperuricemia [Title/Abstract] |
| 11 | gout* [Title/Abstract] |
| 12 | tophus [Title/Abstract] |
| 13 | tophi [Title/Abstract] |
| 14 | uratoma [Title/Abstract] |
| 15 | gophus [Title/Abstract] |
| 16 | tophaceous [Title/Abstract] |
| 17 | "Gout" [Mesh] |
| 18 | "Hyperuricemia" [Mesh] |
| 19 | Acute disease [All Fields] |
| 20 | Acute [All Fields) |
| 21 | or/10-18 |
| 22 | 19 or 20 |
| 23 | 21 and 22 |
| 24 | exp Anti-Inflammatory Agents [Title/Abstract] |
| 25 | exp Anti-Inflammatory Agents, Non-Steroidal [Title/Abstract] |
| 26 | NSAIDs [Title/Abstract] |
| 27 | Aspirin [Title/Abstract] |
| 28 | aspirin [Title/Abstract] |
| 29 | flufenamic acid [Title/Abstract] |
| 30 | mefenamic acid [Title/Abstract] |
| 31 | tolfenamic acid [Title/Abstract] |
| 32 | ibuprofen [Title/Abstract] |
| 33 | ketoprofen [Title/Abstract] |
| 34 | fenoprofen [Title/Abstract] |
| 35 | oxaprozin [Title/Abstract] |
| 36 | sulindac [Title/Abstract] |
| 37 | flurbiprofen [Title/Abstract] |
| 38 | diclofenac [Title/Abstract] |
| 39 | naproxen [Title/Abstract] |
| 40 | tenoxicam [Title/Abstract] |
| 41 | piroxicam [Title/Abstract] |
| 42 | droxicam [Title/Abstract] |
| 43 | Indomet acin [Title/Abstract] |
| 44 | feprazone [Title/Abstract] |
| 45 | phenylbutazone [Title/Abstract] |
| 46 | isoxicam [Title/Abstract] |
| 47 | meclofenamate [Title/Abstract] |
| 48 | ketorolac [Title/Abstract] |
| 49 | lornoxicam [Title/Abstract] |
| 50 | etoricoxib [Title/Abstract] |
| 51 | celecoxib [Title/Abstract] |
| 52 | meloxicam [Title/Abstract] |
| 53 | lumiracoxib [Title/Abstract] |
| 54 | etodolac [Title/Abstract] |
| 55 | nimesulide [Title/Abstract] |
| 56 | exp Cyclooxygenase 2 Inhibitors [Title/Abstract] |
| 57 | exp Cyclooxygenase Inhibitors [Title/Abstract] |
| 58 | cyclooxygenase 2 inhibitor$ [Title/Abstract] |
| 59 | cox 2 inhibitor$ [Title/Abstract] |
| 60 | interleukin-1 [MeSH] |
| 61 | interleukin-1 [Title/Abstract] |
| 62 | Il-1 [Title/Abstract] |
| 63 | “interleukin 1 Receptor Antagonist Protein”[Mesh] |
| 64 | anakinra [Title/Abstract] |
| 65 | canakinumab [Title/Abstract] |
| 66 | canakinumab [Title/Abstract] |
| 67 | rilonacept [Title/Abstract] |
| 68 | rilonacept [Title/Abstract] |
| 69 | ilaris [Title/Abstract] |
| 70 | kineret [Title/Abstract] |
| 71 | arcalyst [Title/Abstract] |
| 72 | “leucocyte pyrogen*” [Title/Abstract] |
| 73 | “leukocytic endogenous mediator*” [Title/Abstract] |
| 74 | “lymphocyte activating factor*” [Title/Abstract] |
| 75 | exp Colchicine [Title/Abstract] |
| 76 | autumn crocus [Title/Abstract] |
| 77 | meadow saffron [Title/Abstract] |
| 78 | colchic$ [Title/Abstract] |
| 79 | colcrys [Title/Abstract] |
| 80 | etoricoxib [Title/Abstract] |
| 81 | arcoxia [Title/Abstract] |
| 82 | glucocorticoid [Title/Abstract] |
| 83 | dexamethasone [Title/Abstract] |
| 84 | prednison [Title/Abstract] |
| 85 | cortisone [Title/Abstract] |
| 86 | hydrocortisone [Title/Abstract] |
| 87 | prednisolone [Title/Abstract] |
| 88 | methylprednisolone [Title/Abstract] |
| 89 | 3-methyl-2-(3-pyridyl)-1-indoleoctanoic acid [Title/Abstract] |
| 90 | allopurinol [Title/Abstract] |
| 91 | apazone [Title/Abstract] |
| 92 | benzbromarone [Title/Abstract] |
| 93 | benziodarone [Title/Abstract] |
| 94 | febuxostat [Title/Abstract] |
| 95 | halofenate [Title/Abstract] |
| 96 | indacrinone [Title/Abstract] |
| 97 | lesinurad [Title/Abstract] |
| 98 | MK 473 [Title/Abstract] |
| 99 | probenecid [Title/Abstract] |
| 100 | pyranoprofen [Title/Abstract] |
| 101 | rasburicase [Title/Abstract] |
| 102 | sulfinpyrazone [Title/Abstract] |
| 103 | Ticrynafen [Title/Abstract] |
| 104 | tisopurine [Title/Abstract] |
| 105 | traxanox [Title/Abstract] |
| 106 | verinurad [Title/Abstract] |
| 107 | zoxazolamine [Title/Abstract] |
| 108 | or/24-107 |
| 109 | 9 and 23 and 108 |
| This search strategy was modified to be suitable for other electronic databases. | |
